# Relationship between person, environmental factors, and activities of daily living performance among physically disabled older adults living at home: a structural equation model

**DOI:** 10.1186/s12877-023-04000-2

**Published:** 2023-05-11

**Authors:** Shuai Fang, Hong Liang, Yan Liang

**Affiliations:** 1 Institute of Sociology, Shanghai Academy of Social Sciences, 622 Huaihai Middle Rd., Huangpu District, Shanghai, 200020 China; 2 School of Social Development and Public Policy, Fudan University, 220 Handan Rd., Yangpu District, Shanghai, 200433 China; 3 School of Nursing, Fudan University, 305 Fenglin Rd., Xuhui District, Shanghai, 200032 China

**Keywords:** Disability, Older adults, Aging in place, Environment, Activities of daily living

## Abstract

**Background:**

Older adults with physical disability need long-term services and support, which incur enormous costs. However, supportive environments may reduce disability and promote aging in place. It is unclear how the physical and social environment affect different types of functional impairments and influence the performance of activities of daily living (ADL) in physically disabled older adults.

**Objective:**

The purpose of this study was to examine the relationship between person, environmental factors, and ADL performance among physically disabled older adults living at home.

**Methods:**

This was a cross-sectional study. Using long-term care insurance claims data from a pilot city in China, we used a structural equation model to assess the potential paths among person, environmental factors, and ADL performance.

**Results:**

Education and income had different influences on the social environment and physical environment. The functional impairments had significant effects on ADL performance, either directly or through physical environment (with handrails) and social environment (family support).

**Conclusions:**

The present findings offer crucial evidence for understanding the interactions between a person and the environment, as well as their influence on physical ADLs, suggesting the importance of a supportive environment and a subpopulation-targeting strategy for disabled older adults.

## Background

The prevalence of disability generally increases with age [1]. Most people aged 80 years and above experience a prolonged period of disability in their final years of life [2]. With rapid population aging worldwide, difficulty with everyday physical functioning—the ability to perform activities of daily living (ADL) may bring massive costs [3]. For example, in United States, an estimated USD 219 billion is spent annually on long- term services and support for functionally dependent individuals [4]. Addressing older adults’ functional goals and home environments may hold promise for reducing disability and advancing aging in place [3].

Environmental perspectives on aging are mainly based on the ecological theory and docility hypothesis [5]. A frequently cited perspective in the literature is the person- environment (P-E) fit [6–8]. Housing Enabler was developed to characterize the P-E fit, using an integrated score to express the magnitude of P–E fit problems for individuals [5, 9]. The definition of behavior as a function of the person and the environment is often considered the basis for P-E fit research [5]. ADL performance is an important aspect of behavior in the research field of P-E fit [10]. However, the P-E process and how their interaction influences individuals’ ADL performance are little understood [5, 7]. Wahl et al. proposed an integrative model to link environment with aging well, suggesting that P-E resources represent the most immediate interface between a person and their environment, which is difficult to disentangle [11].

Models of disability also incorporate the environment, such as the International Classification of Functioning, Disability, and Health (ICF) [12], and the Disablement Process [13], which contribute to better understanding complex P-E relationships. In addition, from the perspective of occupational therapy, the person-environment- occupation (PEO) model is widely used to describe the dynamic interaction between a person, their environment, and occupations [14]. In the context of the PEO framework, the person is seen holistically as a set of attributes (performance components) and life experiences; occupations are defined as personally meaningful activities that individuals’ need, want, or must do as part of their daily life [15]. This framework provides guidance for exploring the relationship between the person, their environment, and occupation (we focused on ADL performance in this study). However, the potential pathways remain unclear, especially considering differences in personal characteristics, functional impairments, physical and social environment.

Previous studies have shown that socioeconomic status (SES) may influence both the physical and social environment. For example, older adults with lower education levels are less likely to have home modifications (HMs) and the impact of income may depend on lower-cost HMs or larger HMs (such as stair lifts) [16]. Social environmental factors are also associated with SES, and recent research not only focuses on older adults in poverty [17] or with low income [18] but also on the broader social environment [19], such as neighborhood attributes (e.g., support and relationships) [20, 21], social connectedness [22], and housing-related policy [23]. Current available evidence also reveals the impact of the physical and social environment on ADL performance [24]. A frequent explanation is that a better physical and social environment increase social participation and engagement in older adults to maintain or enhance ADL performance [21]. However, only a few studies have considered the context of individuals with disabilities [19, 22], and we know little about how the physical and social environment can mediate the link between personal factors (such as SES and different types of functional impairments) and disability (ADL performance).

**Fig. 1:**
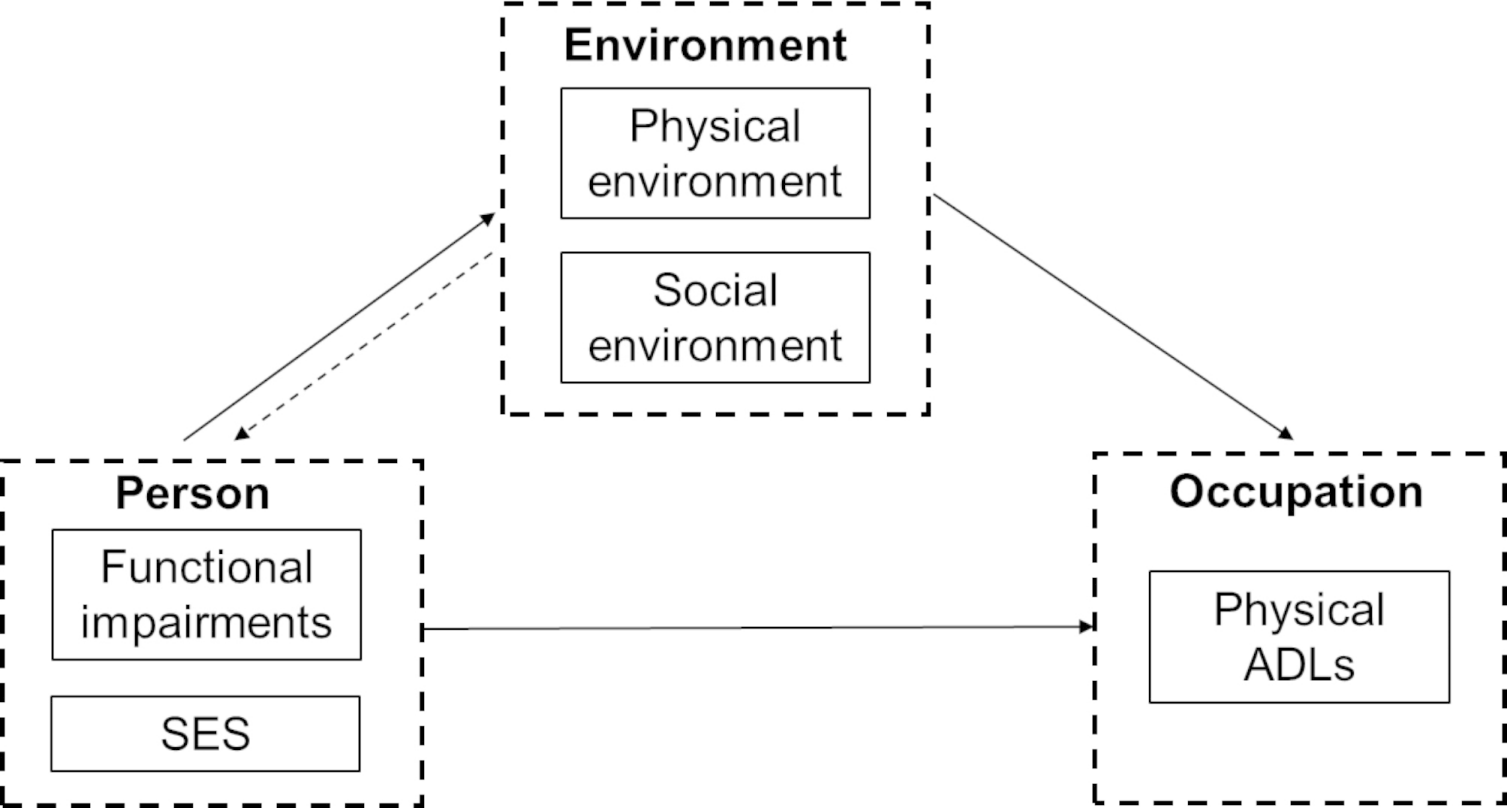
Conceptual framework

To better understand the relationship between the person, environmental factors, and ADL performance among physically disabled older adults living at home, under the guide of the ICF, we proposed a conceptual framework (Fig. 1) combining the model of the disablement process and the PEO model. Based on the disablement process [13] and the PEO model [14], impairment and environmental factors interplay, subsequently influencing disability. Meanwhile, according to the ICF model, individual impairment is a direct result of malfunctions of the body and its structure, not much susceptible to environmental intervention (so this study does not focus on the dotted line in Fig. 1). However, a supportive environment (both physical environment factors and social environment factors) may act as a buffer to mediate the effects of impairment on ADL performance. Several studies, for example, have been carried out on the mediatoring role of the environment. Lee et al. [25] reveal that environmental supports mediate the relationship between functional impairments and psychosocial outcomes (stress) in individuals with multiple sclerosis; Zhang and Li [26] explore how the urban neighborhood environment (such as physical and natural aspect, as well as social aspect) affects quality of life of community-dwelling older adults and develop a mediation model. However, little is known about the mediation path of environmental factors (including physical environment factors and social environment factors) between a person (both SES and functional impairments) and his or her physical ADL performance.

From the perspective of P-E fit [5], as well as the integrative model linking environment with aging well [11], we assume and expect that a person is adaptive to environment to achieve better P-E resources and P-E fit. A deep understanding of the potential mechanism will provide implications to develop tailored intervention to help a person achieve better P-E fit, so as to benefit their ADL performance. Thus, in this study, we focus on the relationship between person, environmental factors, and ADL performance (see the solid lines in Fig. 1), especially the potential mediating roles of environmental factors between personal factors and disability. A structural model was used to test the relationship among person factors (including SES and functional impairments) and environmental factors (both physical environment factors and social environment factors), and their effects on ADL performance. Furthermore, we used long-term care insurance claims data in a pilot city of China to examine the proposed potential paths. We used the results to inform practice and policy makers in developing tailored intervention and strategies to reduce disability.

## Methods

### Data sources and participants

This was a cross-sectional study. We analyzed data from the public Long-term Care Insurance (LTCI) database of Yiwu, Zhejiang Province, China. Yiwu is one of the pilot cities of the long-term care social insurance system in China. All registered population are eligible to participate in the public LTCI. The policy target population is mainly physically disabled older people in the current stage but will expand to the intellectual disabled and will cover all ages in the future. Benefits eligibility is determined via disability criteria, without considering income or assets. A set of standardized assessments was administered by trained professionals who visited claimants’ homes or facilities to determine the qualification of being an LTCI beneficiary. The basic information and physical function status was assessed with a set of survey items by professionals (including doctors and nurses from community health centers), based on the participant’s self-reported information. The database has been described previously [27]. The study population was restricted to adults aged ≥60 years living at home who received the LTCI qualification assessment from September through December 2018. We used deidentified data including information on home environment (both physical and social environment), impairments, physical disability, chronic diseases, and sociodemographic characteristics. In total, 1,675 older adults were included in the analysis. This study was approved by the Institutional Review Board of the researchers’ university (reference number: IRB#TYSQ 2021-12-6).

### Variables

According to the conceptual framework used in this study, we focused on two aspects of person factors: SES and functional impairments. SES includes educational attainment and income, which are widely used indicators of SES [28]. Educational attainment was created as a dichotomized variable based on the distribution (illiterate = 0, primary school and more = 1). Income is a binary variable and coded as low income = 1, other = 0. Low income in the dataset represents those who were certified as a low-income population by the local government.

Functional impairments consisted of mobility impairment, incontinence, and vision impairment [27]. Vision impairment was chosen as an observed indicator and measured using a 5-point scale, with 1 representing vision basically normal and 5 representing severe vision impairment [27]. Incontinence and mobility impairment were generated as latent factors using items chosen from the dataset; the assessment scales have been reported in previous research [27]. The higher the score, the more severe the impairment.

Home environment factors consisted of physical environment factors and social environment factors. Based on prior literature and the dataset, physical environment was measured using four factors and social environment was measured using three factors in this study. For physical environment, we chose handrails [29], without steps [30], access to a lift (or living on the first floor) [31], and a private toilet/bathroom [32] as observed indicators and coded them as binary variables. Participants who reported having handrails, without steps, living on the first floor or living on the second floor or above but having access to a lift, or having a private toilet/bathroom indoors, were coded as “1” for the above mentioned four observed indicators, respectively; otherwise, they were coded as “0”. For social environment, we chose one observed indicator and generated one latent factor. Living with family members was used as an observed indicator and coded as “1” for responses of “yes”; otherwise, these were coded as “0”. Family support was created as a latent factor based on the two self-reported items from the dataset: receiving family emotional support (yes = 1, no = 0), and receiving family material support (yes = 1, no = 0).

Eight items were chosen to assess ADL performance among participants, such as eating, brushing, and washing [27]. The measurement level of each item was 1–5 levels of dependency, ranging from 1 (not needing assistance) to 5 (needing full assistance).

Confirmative factor analysis (CFA) was performed to generate factor scores of family support, incontinence, mobility impairment, and ADL performance.

Age, sex, marital status, and physical health status were considered as confounders and were measured as follows: (1) age (years); (2) sex (male = 0, female = 1); (3) marital status (married = 0, and other = 1); and (4) having any chronic disease (no = 0, yes = 1).

### Statistical analyses

Descriptive statistics were used to summarize sample characteristics. Structural equation modeling (SEM) was used to examine the relationship between person and environmental factors, and ADL performance. Confirmative factor analysis was used to verify the structure validity of latent variables, namely, family support, incontinence, mobility impairment, and ADL performance. The four latent variables were measured using the indicators, as described in the Variables section. We used regression models to estimate the association between person and environmental factors, and ADL performance, adjusting for demographic and health variables. We performed log transformation of each individual’s age given its distribution and used the standardized variable in the SEM. As several dependent variables are categorical in the SEM, a weighted least squares estimate was used [33]. This estimation method, also referred to as a robust weighted least squares (WLS) approach in the statistics literature, is referred to as WLSMV, for weighted least squares mean and variance adjusted in Mplus. The WLSMV approach seems to work well if sample size is 200 or better [34]. Model fit was assessed using the following indexes: comparative fit index (CFI) > 0.9, Tucker–Lewis index (TLI) > 0.9, root mean square error of approximation (RMSEA) < 0.08, standardized root mean square residual (SRMR) < 0.05, and chi-square/degrees of freedom (df) < 5.0. All analyses were performed with Mplus 8.0 and Stata SE 17.0.

## Results

### Participant characteristics and home environment

Table 1 presents characteristics of participants and their home environment. The mean age of the sample was 78.2 years. Nearly half (49.3%) of participants were women, and 82.0% were married. Most (75.2%) participants reported having chronic diseases. About half (51.1%) participants reported an educational level of illiterate, and 13.3% had low-income levels. Most (76.6%) participants reported that they were living with family members. For physical environment, only 7.2% of participants reported having handrails, 27.9% had access to a lift (or living on the first floor), 81.6% reported not having steps, and 90.4% had a private toilet/bathroom.

**Table 1:** Participant characteristics and home environment (*N* = 1675)

Variable	n (%) or M ± SD	
**Age (years)**		78.2 ± 9.37
**Sex**		
Female		826 (49.3)
Male		849 (50.7)
**Marital status**		
Married		1,373 (82.0)
Other		302 (18.0)
**Chronic disease**		
Yes		1,260 (75.2)
No		415 (24.8)
**Education**		
Illiterate		856 (51.1)
Primary school and above		819 (48.9)
**Income**		
Low income		223 (13.3)
Higher income		1,452 (86.7)
**Social environment**		
Living with family members (living status)		
Yes		1,283 (76.6)
No		392 (23.4)
**Physical environment**		
With handrails (yes)		120 (7.2)
Without steps (yes)		1,367 (81.6)
Access to a lift/living on the first floor (yes)		468 (27.9)
Having a private toilet/bathroom (yes)		1,514 (90.4)

### Measurement model

CFA was performed to confirm an acceptable fit of the latent variable constructs: mobility, incontinence, physical ADLs, and family support. CFA showed good fit indices for the four latent variables: chi-square/df = 2.47, p < 0.001, CFI = 0.994, TLI = 0.990, RMSEA = 0.030, and SRMR = 0.021, after model re-specification by correlating error terms according to empirical rationales. The standardized factor loadings of the observed variables ranged from 0.664 to 0.967 (Table 2).

**Table 2:** Confirmatory factor analysis and factor loadings

Four factors and scale items	Standardized loading
Mobility	
Turning over in the lying position	0.835
From sitting to standing	0.902
Maintain sitting in a chair	0.809
Walk (move) about 5 m on the flat floor	0.809
Maintain balance	0.750
Upstairs and downstairs	0.702
Incontinence	
Urinary incontinence	0.967
Fecal incontinence	0.932
Physical ADLs	
Eating	0.873
Tooth and hair brushing	0.759
Washing	0.763
Grooming	0.699
Putting on clothes	0.804
Putting on pants	0.837
Bathing	0.745
Toileting	0.779
Family support	
Emotional support	0.940
Material support	0.664

Notes: All standardized factor loadings are significant at p < 0.001

### Structural model

**Fig. 2:**
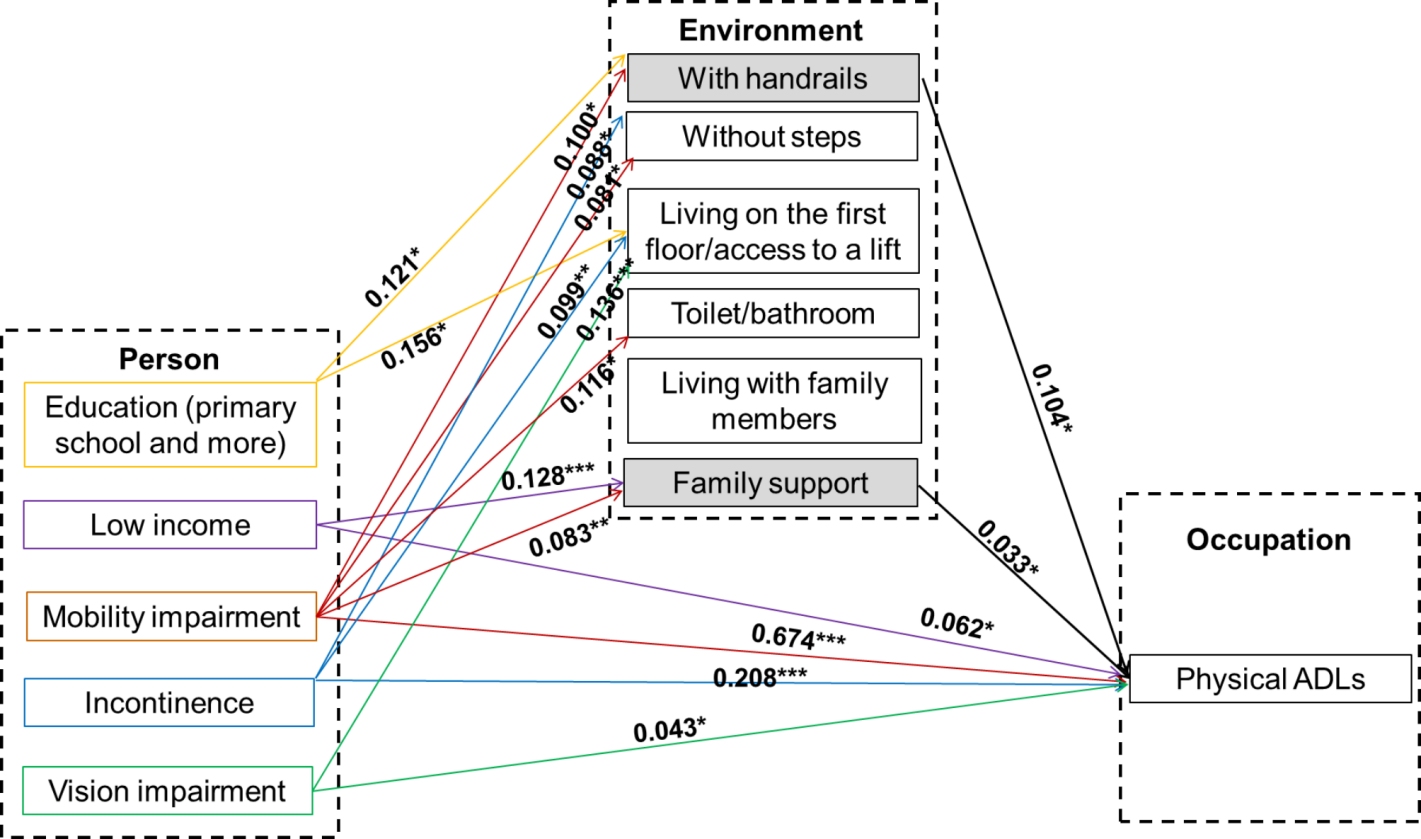
Results of structural equation model

After identifying a well-fitted measurement model, the relationships between all variables in the structural model were tested. The results of the structural model showed a good fit for the data (chi-square/df = 2.79, p < 0.001, CFI = 0.93, TLI = 0.90, RMSEA = 0.033, and SRMR = 0.044) (Fig. 2).

The results of the structural model showed that education and income had a significant influence on both the social environment and the physical environment. Participants with higher education (primary school and more) were more likely to have a better physical environment (with handrails or access to a lift/living on the first floor); while those with low income were more likely to have better family support. The functional impairments had significant effects on ADL performance, either directly or through physical environment (with handrails) and social environment (family support). Physical environment (with handrails) and social environment (family support) mediate the association between personal factors and ADL performance.

Specifically, Mobility impairment was positively and significantly correlated with having handrails, without steps, having a private toilet/bathroom indoors, family support, as well as physical ADLs; incontinence was positively and significantly related to without steps, having access to a lift/living on the first floor, and physical ADLs; vision impairment was positively and significantly associated with having access to a lift/living on the first floor and physical ADLs. With handrails and family support were positively and significantly associated with physical ADLs.

## Discussion

Using long-term care insurance claims data from a pilot city in China, through the lens of the P-E-O framework and disablement process, we explored and identified potential pathways among person, environmental factors, and ADL performance in a unique sample of physically disabled older adults living at home. Physical environment (with handrails) and social environment (family support) mediate the association between personal factors and ADL performance; however, no potential buffering effect of environmental factors was found. Nevertheless, the findings offer crucial evidence for understanding the relationship between person and environment, as well as their influence on physical ADLs, suggesting the importance of a supportive environment and a subpopulation-targeting strategy for disabled older adults.

In terms of physical environment, with handrails mediates the link between mobility impairment and ADL performance. This may be understood from the perspective of P-E fit and lends support to previous findings [5, 7, 8]. For older adults with mobility impairment, handrails provide balance assistance, thus affecting their ADL performance. In our study, other physical environment factors related to hygiene (a private toilet/bathroom) and transfers (no steps and access to a lift/living on the first floor) are not significantly associated with physical ADLs. The possible reason is that our study population, the LTCI claimants, are more likely to be home bound even bed bound people. This suggests that an early-stage environment intervention is needed to provide potentially disabled people better P-E fit.

Although the potential mediating roles of other physical environment factors related to hygiene and transfers are not found between functional impairments and ADL performance in our study, functional impairments are significantly associated with both these physical environment factors and ADL performance. Older adults with functional impairments are more likely to have environment intervention, such as having handrails, better hygiene and transfer conditions. The findings are consistent with previous research. For example, older adults with incontinence have caused a considerable burden of informal care[35], and family members affected by incontinence care are more likely to adapt to the physical environment to reduce transfer obstacles, so as to ease the care burden [16, 36].

In addition, such positive association between functional impairments and environment intervention can be explained through the lens of P-E interchange [11]. According to the integrative model of healthy aging and the environment [11], experience-driven belonging and behavior-driven agency represent two processes of P-E interchange, and the core of these two processes is P-E resources. Specifically, better P-E resources lead to a greater feeling of belonging, which reflects a sense of positive connection with other people and the environment; as well as better agency, which means becoming an agent of change through proactive behaviors [11]. Environment intervention is a kind of such proactive behaviors for both disabled older adults and their family members, so as to pursue better P-E resources.

Social environment (family support) can mediate the links between personal factors (low income/mobility impairment) and ADL performance. Older adults with low income or more severe mobility impairment are more likely to have better family support. A possible explanation is that older adults with low incomes have fewer options and are more dependent on family support than those with higher incomes. Older adults with higher incomes may have more choice of living arrangements, such as receiving care from domestic workers [37] or in care institutions; these individuals may also have greater choice in migration, such as from rural to urban areas, which weakens family-based care. For example, Cao et al. found that healthier older people with a higher income/education are more likely to migrate in China [38]. Additionally, this positive association between mobility impairment and family support may be explained in the context of Chinese culture. Traditional Chinese culture values filial piety and family care [39]; thus, older people with impaired mobility may have more family support. Previous studies have highlighted the roles of social support [20], and perceived “respect and social inclusion” on mobility [40]. Our findings contribute to this research field by providing new evidence in the context of Chinese culture, and suggest that family members are vital in enhancing a supportive social environment.

Our study provides an opportunity to understand the association between SES and the home environment, especially within the context of welfare policy. Education and income showed different effects on physical and social environments. Older adults with higher education levels were more likely to have a better physical environment; those with lower incomes were more likely to have better family support. Such disparity has also been reported in previous studies [16, 41]. Our findings suggest that China’s welfare policies should be more targeted to subpopulations in order to alleviate such disparity. For example, more home modification programs are provided to not only older people below the safety net, but also those with lower education; Providing respite services to low-income people to support their family care. According to reports of the State Council of China, 164,000 households including older adults living in extreme poverty received home modification during the “13th Five Year Plan” period (2016–2020) [42]. Our findings show that illiterate older adults were less likely to have home modification. This may be because China’s age-friendly community policy is still in progress.

Several limitations of this study should be noted. First, this study followed a cross-sectional design, which precludes inference regarding cause-and-effect relationships among the studied factors. For example, we find that older adults having handrails or having more family support are associated with higher physical dependency. As the present cross-sectional design precluded inference on causality, we are cautious about the results that no potential buffering effect of environmental factors is found. Additional longitudinal studies are needed to identify the potential mechanism. Second, this was a secondary analysis using public long-term care insurance claimant data, and some physical environment factors, as well as functional impairment (such as hearing impairment) were not included due to data limitation. Third, we only enrolled participants from one city in eastern China, which limits the generalizability of the results to other areas of China.

Despite the above limitations, the present findings have strong implications for practice and policy. First, a supportive home environment must be considered an important part in the development of a long-term service and support system for disabled older adults. Home modification services should be expanded from the most vulnerable to the entire population of older adults, contributing to delaying potential functional limitation during aging. Furthermore, a subpopulation-targeting strategy is needed. Tailored intervention should be developed considering specific personal characteristics of older adults who have low incomes, or those who are illiterate, to develop better P-E resources to support independence and reduce the burden of care.

## Conclusions

The effects of education and income on the social environment and physical environment were quite different. Different physical and social environment factors showed different paths of influence on the relationship between personal factors and ADL performance. Future research is needed to further explore the complexity of person–environment interaction and their influence on ADL performance using a longitudinal design. Our study suggests the direction of future efforts, which should consider SES and the physical and social environment of older adults to build a supportive environment and adopt a subpopulation-targeting strategy.

## Data Availability

The publication of the data in a public data repository is not possible due to the restriction of data management. The data can be accessed from the corresponding author on reasonable request.
